# Mn-Based MRI Contrast Agents: An Overview

**DOI:** 10.3390/molecules28217275

**Published:** 2023-10-26

**Authors:** Céline Henoumont, Marie Devreux, Sophie Laurent

**Affiliations:** 1 NMR and Molecular Imaging Laboratory, Department of General, Organic and Biomedical Chemistry, University of Mons, 19 Avenue Maistriau, 7000 Mons, Belgium; celine.henoumont@umons.ac.be (C.H.);; 2 Center for Microscopy and Molecular Imaging (CMMI), 8 Rue Adrienne Boland, 6041 Gosselies, Belgium

**Keywords:** Mn complexes, Mn-based nanoparticles, theranostic, magnetic resonance imaging

## Abstract

MRI contrast agents are required in the clinic to detect some pathologies, such as cancers. Nevertheless, at the moment, only small extracellular and non-specific gadolinium complexes are available for clinicians. Moreover, safety issues have recently emerged concerning the use of gadolinium complexes; hence, alternatives are urgently needed. Manganese-based MRI contrast agents could be one of these alternatives and increasing numbers of studies are available in the literature. This review aims at synthesizing all the research, from small Mn complexes to nanoparticular agents, including theranostic agents, to highlight all the efforts already made by the scientific community to obtain highly efficient agents but also evidence of the weaknesses of the developed systems.

## 1. Introduction

**Figure 1:**
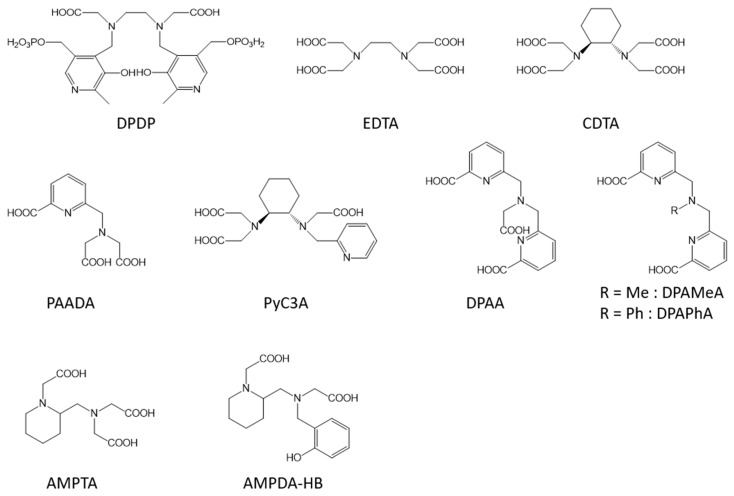
Linear ligands discussed in this work.

Medical diagnosis by magnetic resonance imaging (MRI) often requires the use of contrast agents to highlight some pathological regions, such as tumor tissues. Gadolinium complexes are currently the most utilized contrast agents in the clinical field thanks to their property of enhancing water proton longitudinal relaxation [1]. Nevertheless, for many years, it has been demonstrated that the injection of gadolinium complexes to patients with kidney failure is responsible for the apparition of a disease called nephrogenic systemic fibrosis (NSF) [2,3,4,5]. It has been particularly evidenced for linear gadolinium complexes based on acyclic ligands such as Gd-DTPA (gadopentetic acid) since they are less thermodynamically and kinetically stable than those based on macrocyclic complexes such as Gd-DOTA (gadoteric acid). Therefore, those acyclic Gd-based CAs (GBCAs) are no longer recommended for those patients [2,3,6]. Additionally, more recent studies have demonstrated an accumulation of gadolinium CAs in the brain of subjects with normal renal function [5,6,7,8,9,10]. This situation has attracted research on Gd-free alternatives, among which we can cite the development of magnetic nanoparticles [11], fluorine MRI [12], non-metal nitroxide radical based systems [13], and manganese-based CAs on which this review will focus [14,15,16]. Manganese ions can be found in the body under the form of Mn^2+^ or Mn^3+^ with five or four unpaired d-orbital electrons, respectively. Its normal physiological concentration in the serum of healthy subjects is about 0.5–1.2 µg/dL (9–22 µM) and it will work in the organism as a cofactor activating some enzymes or as a constituent in metalloenzymes. Manganese ions also act in the development of the immune and nerve system functions and in the regulation of vitamins and sugar in the blood [17,18]. Mn-based MRI contrast agents were first used as an oral formulation containing liposome-encapsulated MnCl_2_ salt (LumenHance^®^), indicated for gastrointestinal images. Nevertheless, it was shown that too high doses of free manganese ions could induce a neurodegenerative disorder called manganism, a disease with symptoms similar to those of Parkinson’s disease. This contrast agent is therefore no longer used but manganese-enhanced MRI (MEMRI) using MnCl_2_ is still utilized for preclinical studies in mice with brain [19] or lung [20] model tumors. For safe use in the clinic, manganese complexes have been developed and the second manganese-based CA approved by the Food and Drug Administration (FDA) in 1997 was manganese dipyridoxyl diphosphate (Mn-DPDP, Teslascan^®^, Figure 1) for use as a liver-specific hepatobiliary CA [21,22]. Nevertheless, its efficacy was quite limited and some toxicity issues were evidenced due to the release of free Mn ions in vivo. As a result, Mn-DPDP is no longer commercialized for clinical use so there is still a need for Gd-free alternatives with high thermodynamic stability and kinetic inertness and with a high efficacy to be competitive with GBCAs [14,15,16,23]. This review aims at synthesizing the developed Mn-based MRI contrast agents since increasing amounts of research is conducted on this subject with a high diversity on the proposed structures but a unique goal: the ability of the Mn-agents to reach their target at a low dose and to produce a sufficiently high MRI signal.

## 2. Molecular Mn-Based Contrast Agents

Similarly to the Gd-complexes, the efficacy of molecular Mn-based contrast agents is based on the presence of at least one exchanging water molecule in the inner coordination sphere of the metal characterized by a fast exchange rate. With the typical coordination number of Mn(II) complexes in aqueous solution being six, seven, or sometimes eight, this innersphere water molecule is assured if the ligand possesses five or six coordination bonds with the metal. Nevertheless, the thermodynamic stability and the kinetic inertness of Mn complexes is generally lower than that of Gd-complexes because of the lower charge of Mn ions and the lack of ligand-field stabilization energy (high spin d^5^ electron configuration). Moreover, the possible oxidation of Mn^2+^ to Mn^3+^, which is often related to the thermodynamic stability of the Mn(II)-complex, also has to be avoided as it will lead to a loss of efficacy because of the loss of one unpaired electron and of a less favorable electronic relaxation. Nevertheless, one example of Mn(III) complexes can be found in the literature [24]. They are based on planar tetradentate chelates assembled from a 1,2-phenylenediamido backbone. Their relaxivity, defined as the increase in the water proton relaxation rate induced by 1 mmole per liter of the contrast agent, is comparable to that of clinically used Gd-based contrast agents; they are moreover able to accumulate in intracellular compartments.

Thus, in their efforts to incorporate manganese ions in a stable and efficacious structure, researchers have developed a lot of different ligands to coordinate Mn ions, even linear or cyclic ligands [14,15,16]. All those developed complexes are non-specific and rapidly cleared by the kidneys or targeted to specific organs or tissues such as tumors or the liver. Some of them can also be responsive to a certain stimulus or combine several imaging techniques. This will be developed in the following, with the exception of a particular class of ligands, the porphyrins, which allow the incorporation of Mn ions in the center of the heme ring. Mn-porphyrins represent a very interesting and promising class of Mn-based contrast agents characterized by high relaxivities but they were the object of a recent extensive review [25] and so they will not be described again here.

### 2.1. Non-Specific Contrast Agents

*Complexes based on linear ligands:*

EDTA (ethylene diamine tetraacetic acid, Figure 1) is a linear hexadentate ligand able to form very stable complexes with Mn ions and was thus extensively studied. Indeed, previous works on [Mn(EDTA)(H_2_O)]^2−^ have shown that its sodium salt is very well tolerated: LD50 is 7.0 mmol/kg in rats following intravenous injection compared with an LD50 of 0.22 mmol/kg for MnCl_2_ [26]. Moreover, it allows the presence of one fast-exchanging water molecule so that the relaxivities of the complexes [Mn(EDTA)(H_2_O)]^2−^ and [Gd(DTPA)(H_2_O)]^2−^ are similar (2.9 and 4.1 mM^−1^ s^−1^, respectively, at 20 MHz, 35 °C) (Table 1) [27]. The increased relaxivity of the Gd(III) complex may be due to its larger size and slower tumbling rate. As it is well-known that a decrease in the tumbling rate can boost the efficacy of the contrast agents, several studies have tried to increase the size of the Mn-complex. We can cite, for example, the work of Caravan et al. [28] who have grafted six tyrosine-derived [Mn(EDTA)(H_2_O)]^2−^ moieties to a cyclotriphosphazene core. The 37 °C per Mn(II) relaxivity ranged from 8.2 to 3.8 mM^−1^ s^−1^ from 0.47 to 11.7 T and is sixfold higher on a per molecule basis. Other research was focused on the improvement in the stability of the Mn complexes based on linear ligands by rigidifying the chelator. We can cite the addition of a cyclohexane ring (Mn-CDTA) [29,30] of one or two pyridine rings (Mn-PyC3A, Mn-PAADA, Mn-DPAA, Mn-DPAMeA, or Mn-DPAPhA) [31,32,33,34,35,36] or of the piperidine rings (Mn-AMPTA or Mn-AMPDA-HB) [37] (Figure 1, Table 1) forming pentadentate or hexadentate ligands. As expected, pentadentate complexes were globally less stable than hexadentate complexes whereas the increased rigidity of the chelator allowed more stable complexes to be obtained. For example, the pMn of Mn-CDTA is higher than that of Mn-EDTA (8.67 for Mn-CDTA versus 7.82 for Mn-EDTA at pH 7.4) [29]. Mn-PyC3A [31,32,33,34] can also be cited for its good thermodynamic (pMn of 8.17 at pH 7.4) and kinetic stability as well as a good relaxivity of 2.1 s^−1^ mM^−1^ at 1.4 T and 37 °C (Table 1); it has recently started phase I clinical trials (NCT05413668). Moreover, it has been demonstrated as a potential alternative to gadolinium to characterize acute myocardial infarctions [38]. In the attempts to increase the stability of the complexes and the kinetic inertness toward endogenous ions such as Zn^2+^ ions, the work of Wadepohl et al. [39] opens interesting perspectives. It is based on bispidine derivatives providing rigid and large coordination cavities that perfectly match the size of Mn^2+^ ions.

All those cited complexes often represent difficult synthetic procedures; the study by Stasiuk et al. [40], who proposed a single-pot template reaction to obtain an Mn-based contrast agent endowed with a good kinetic inertness toward zinc transmetallation, as well as an interesting relaxivity of 5.2 s^−1^ mM^−1^ at 1.5 T and 298 K, is thus interesting.

*Complexes based on macrocyclic ligands:*

Globally, complexes based on macrocyclic ligands are more thermodynamically stable than those based on linear ligands, hence, a lot of research is focused on macrocyclic complexes.

**Figure 2:**
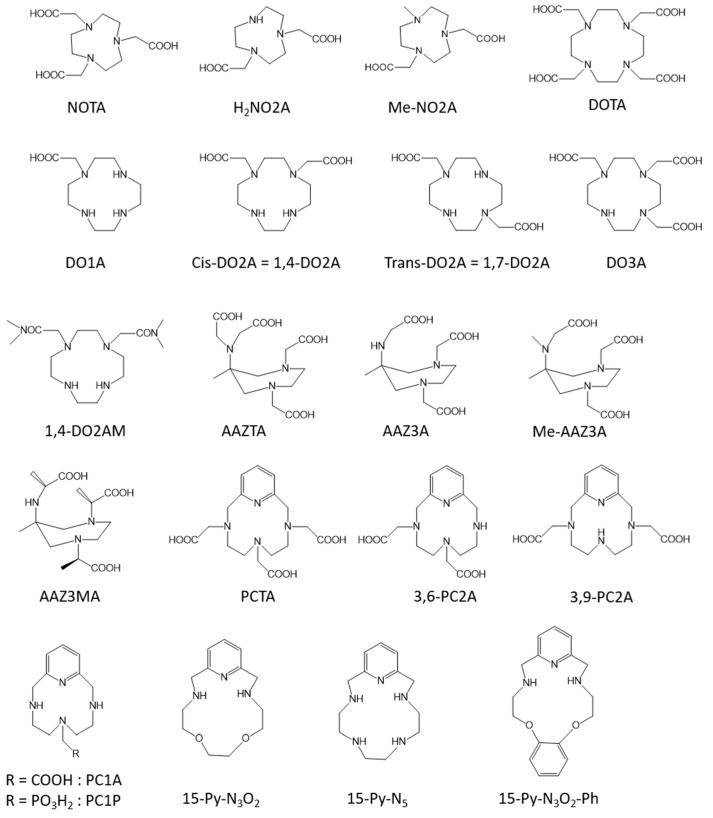
Structure of the macrocyclic ligands discussed in this work.

The first well-known category of macrocyclic ligands are the triazacyclononane derivatives. Several studies have shown that hexadentate ligands, such as NOTA (Figure 2) [41] or its derivatives where one acetate pendant arm is replaced by other donor groups such as a sulfonamide [42], an acetamide [43], or a methylene pyridine group [44], lack the presence of one innersphere water molecule when they are complexed with Mn(II) ions, with consequently very low relaxivities. Rodriguez-Rodriguez et al. [43] took advantage of this absence of any innersphere water molecule to more thoroughly study the effect of the electron spin relaxation at a low field and they have shown that the electronic relaxation is quite insensitive to the nature of the donor atom but depends more on the coordination polyhedron. Nevertheless, if one of the donor groups is replaced by other substituents, pentadentate ligands (1,4,7,-triazacyclononane-1,4-diacetic acid, H_2_NO_2_A, Figure 2, Table 1) allowing the presence of one innersphere water molecule when complexed to Mn(II) ions are obtained. Those complexes were extensively studied [44,45,46,47,48] with a special interest in the more recent study [48] on the water exchange rate, which has to be sufficiently high to ensure good relaxivity. The authors have used ^17^O measurements and DFT calculations to establish that the water exchange rate is greatly influenced by the bulkiness of the substituent at position seven of the triazacyclononane unit.

Similarly to NOTA, the DOTA ligand (Figure 2), a well-known tetraazatetradecane ligand, does not allow the presence of one innersphere water molecule when complexed to Mn(II) ions. Nevertheless, as Toth et al. [41] have shown a highest kinetic stability toward zinc transmetallation for the Mn-DOTA complex compared to the Mn-NOTA, derivatives of DOTA allowing the presence of one innersphere water molecules could be interesting to investigate. Therefore, Mn(II) complexes with cyclen-based ligands bearing one, two, and three acetate pendant arms [49,50] (DO1A = 1,4,7,10-tetraazacyclododecane-1-acetic acid, cis- and trans-DO2A (Cis = 1,4,7,10-tetraazacyclododecane-1,4-diacetic acid, Trans = 1,4,7,10-tetraazacyclododecane-1,7-diacetic acid), and DO3A = 1,4,7,10-tetraazacyclododecane-1,4,7-triacetic acid, Figure 2) were studied by ^1^H and ^17^O relaxometry. The results were the absence of any innersphere water molecule for Mn-DO3A, as well as for Mn-trans-DO2A, whereas Mn-cis-DO2A and Mn-DO1A complexes contain one innersphere water molecule (Table 1). It nevertheless has to be noted that the decreased denticity of the ligand, as expected, results in a decrease in the complex stability. Botta et al. [51] also investigated the replacement of acetate by N,N-dimethylacetamides pendant arms (1,4-DO2AM, Figure 2, Table 1) and they obtained an increased kinetic inertness. This was confirmed by the study of Garda et al. [52] who replaced the acetate arms by phosphonate arms or mono-, secondary-, or tertiary amides arms and their results point out that phosphonates lead to a decrease in the complex stability whereas tertiary amides afforded encouraging results to increase the stability.

The AAZTA ligand (Figure 2) [29] (AAZTA = 6-amino-6-methylperhydro-1,4-diazepine tetraacetic acid) is another macrocyclic chelate able to complex Gd^3+^ and Mn^2+^ ions. Similarly to the case of DOTA, whereas the Gd-AAZTA complex allows the presence of two innersphere water molecules, the Mn-AAZTA complex is characterized by the absence of water co-ligand; hence, its relaxivity is quite low. Botta et al. [53] have thus synthesized three AAZTA derivatives with only three acetate or α-methylacetate arms (Mn-AAZ3A, Mn-MeAAZ3A, and Mn-AAZ3MA, Figure 2). Those complexes have one innersphere water molecule and hence a better relaxivity (Table 1) but once again to the detriment of the stability (huge decrease in the pMn value for the three derivatives compared to Mn-AAZTA).

Pyclen (3,6,9,15-tetraazabicyclo[9.3.1]pentadeca-1(15),11,13-triene) is another interesting 12-membered macrocyclic structure characterized by an *N*-pyridyl donor that rigidifies and pre-organizes the ligand coordinating groups (in particular rendering the four nitrogen atoms coplanar) which could improve the kinetic inertness of the resulting complex. The pyridine subunit also endows the ligand with an increased degree of lipophilicity that could induce mixed renal and hepatobiliary clearances, another interesting advantage in the context of patients with reduced kidney function. The pyclen [12] PyN4) macrocyclic core is now recognized to form efficient chelators for the Mn^2+^ cation complexation and some studies can be found on the interest of such pyridine-containing (PC) ligands. Garda et al. [52] studied derivatives of PCTA (Figure 2) with three pendant arms and studied the influence of the presence of a primary, a secondary, or a tertiary amide instead of the carboxylate functions on the proton relaxometry, the thermodynamic stability, and the kinetic inertness. Similarly to their results on DOTA derivatives, the presence of tertiary amides as pendant arms allows an increase in the stability of the complexes. However, the corresponding Mn(II)-complexes show quite low relaxivities, of less than 2 s^−1^·mM^−1^ at 37 °C and 20 MHz, due to the absence of any innersphere water molecule. To increase the relaxivity, the denticity of the ligand has to be decreased and derivatives of PC2A (with two pendant arms) were developed. We can cite the development of 3,6-PC2A and 3,9-PC2A depending on the position of the two acetate pendant arms (Figure 2) [54]. The authors found better complex stability for the 3,9-PC2A complex compared to 3,6-PC2A, with both complexes having one innersphere water molecule and being characterized by relaxivities similar to that of the clinically used Gd-DOTA (r_1_^p^ = 2.72 and 2.91 mM^−1^ s^−1^ for the complexes 3,6-PC2A and 3,9-PC2A, respectively, at 25 °C and 0.47 T, Table 1). Laurent et al. [55] also developed three derivatives of 3,9-PC2A including an additional function grafted onto the pyridine ring to allow conjugation to a molecule of interest. Those three complexes are endowed with one innersphere water molecule and exhibit similar relaxivities to that of the 3,9-PC2A complex. Drahos et al. [56] again decreased the denticity of the ligand by studying derivatives with one pendant arm (PC1A, Figure 2). They evaluated the influence of the nature of the coordinating group of this arm (acetate versus methylphosphonate group) on the thermodynamic stability, the kinetic inertness, the redox potential, and the ^1^H and ^17^O relaxation. They found that those mono-functionalized pyclens PC1A and PC1P give ternary hexacoordinate Mn^2+^ complexes which accommodate one water co-ligand, both of them being very labile and undergoing oxidation to the Mn^3+^ form, proving once again that the decrease in the denticity of the ligand is detrimental to the stability of the complexes.

Pyridine-based 15-membered macrocyclic ligands were also developed to complex manganese ions. Drahos et al. [57] developed Mn-15-pyN5 and Mn-15-pyN_3_O_2_ complexes (Figure 2). They obtained a good thermodynamic and kinetic inertness, especially for Mn-15-pyN5, where the two additional nitrogens allowed a higher thermodynamic stability; but, this stability is nevertheless too low for in vivo applications. Moreover, their relaxivities were quite high thanks to the presence of two innersphere water molecules (Table 1). Green et al. [58] also developed the same kind of complexes with an additional ortho-phenylene unit (Mn-15-pyN_3_O_2_-Ph, Figure 2) but the stability was rather low so it is unsuitable as an MRI contrast agent. More recently, Drahos et al. [59] added an additional acetate pendant arm to those pyridine-based 15-membered ligands in order to increase the stability and the kinetic inertness as well as the solubility in water. The results show a decrease in the relaxivity compared to Mn-15-pyN5 and Mn-15-pyN_3_O_2_ complexes since the presence of the additional acetate pendant arm leads to a decrease in the number of innersphere water molecules from two to one. The kinetic inertness is, however, slightly better but remains quite low for in vivo applications.

Recently, Mayilmurugan et al. [60] reported the design of new phenylenediamine-based macrocyclic ligands to complex Mn(II) ions. Their results show good thermodynamic and kinetic inertness as well as interesting relaxivities typical of complexes characterized by one water co-ligand so they could be promising for future use as MRI contrast agents. We can also cite the study of Boschi et al. [61] who reported the development of a new class of Mn(II)-dithiocarbamates complexes. They obtained relaxivities similar to those of Gd-DOTA but the stability tests have still to be conducted.

Globally, it can be evidenced that the thermodynamic stability and the kinetic inertness of the obtained Mn complexes remains a major issue. Esteban-Gomez et al. [62] tried to analyze this stability using structural descriptors and evidenced some donor groups particularly suited to form stable chelates at physiological pH, such as 2-methylpyridine, secondary and tertiary acetamide or picolinate groups. A lot of efforts are, however, still to be performed to obtain highly stable Mn complexes with good relaxivity, the most promising complexes appearing to be Mn-CDTA and its derivatives, Mn-PyC3A, Mn-PC2A and its derivatives, and Mn-1,4-DO2A and its derivatives.

### 2.2. Liver Targeted Contrast Agents

**Figure 3:**
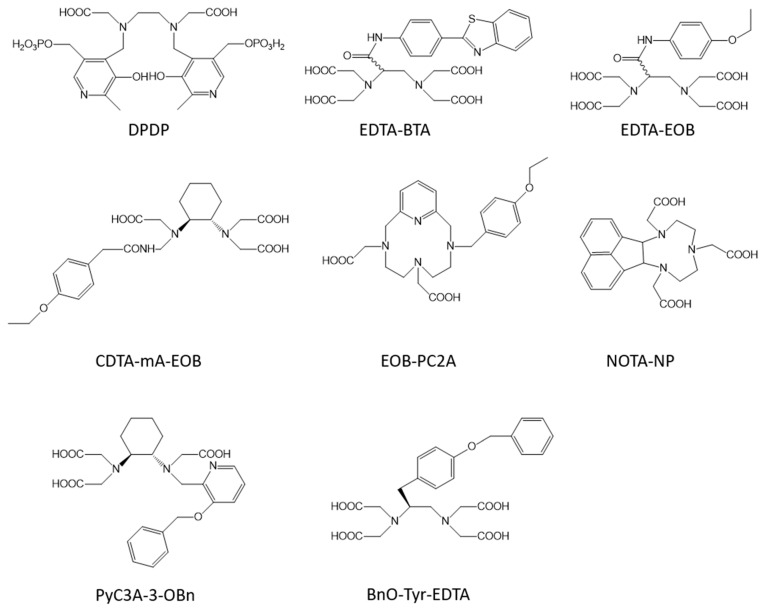
Structure of some ligands used to construct liver-targeted contrast agents.

The development of liver-targeted MRI contrast agents has a double objective: first, it can allow the diagnosis of liver diseases such as tumors and secondly, elimination through the liver instead of the kidneys could be safer for patients suffering from a kidney chronic disease. Mn-DPDP (Figure 3) [21,22] was the first clinically used Mn complex as liver targeted MRI contrast agent but it is no longer used because of its low relaxivity (2.8 s^−1^·mM^−1^ at 20 MHz and 40 °C) due to the lack of any innersphere water molecule, and toxicity issues due to the low thermodynamic stability of the complex, which releases free Mn ions in vivo. Therefore, the development of those agents remains an important challenge.

Human organic anion transporting polypeptides (OATPs), expressed in functioning hepatocytes, can induce the cellular uptake of several amphiphilic organic molecules, such as bile salts, bilirubin, steroid hormones, thyroid hormones, and so on. Therefore, the development of amphiphilic Mn complexes, bearing a lipophilic group on the chelate to mediate an uptake by the liver, is well-studied in the literature. Moreover, it has to be noted that a compromise has to be found between a sufficient lipophilicity to promote an avid hepatobiliary accumulation and a rapid blood clearance to allow a fast diagnosis. Indeed, it has been shown previously that an increased lipophilicity can also promote binding to serum proteins, such as albumin, which will prolong the blood circulation time. The following Mn complexes have shown interesting properties: Mn-EDTA-BTA [63], where the lipophilicity is provided by a benzothiazole aniline grafted on the EDTA coordination cage; complexes incorporating the well-known EOB (ethoxybenzyl) moiety, already used on Gd complexes, on EDTA [64] (Mn-EDTA-EOB), on CDTA [65] (Mn-CDTA-mA-EOB), and on PC2A [66] (Mn-EOB-PC2A); Mn-NOTA-NP [67], where a naphthalene group is grafted on NOTA; Mn-PyC3A-3-OBn [68], where a benzyloxy group is grafted at position 3 of the pyridine group of the chelator PyC3A; or Mn-BnO-TyEDTA [69,70], where the lipophilicity is also provided by a benzyloxy group introduced on the backbone of tyrosine-derived Mn-EDTA (Figure 3). All those agents are endowed with a similar relaxivity comprised between 2.5 and 3.5 s^−1^ mM^−1^ at 1.5 T and 298 K (Table 1) and undergo partial renal and hepatobiliary excretion.

More specifically, a Mn complex has recently been developed to image liver fibrogenesis. This pathology is accompanied by the upregulation of lysyl oxidase enzymes, which causes the apparition of aldehyde-containing amino acid allysine (Lys^Ald^) on the extracellular matrix proteins. A series of stable hydrazine-equipped manganese MRI probes able to bind to those modified proteins were thus developed, with promising results [71].

### 2.3. Blood Pool Agents

Blood pool agents are characterized by a long vascular circulation time so that they can be used for vascular imaging. MR hardware now enables high-quality vascular images to be recorded with extracellular agents a few seconds after the injection so that the development of blood pool agents appears less important [32]. Nevertheless, they could still be interesting for some specific applications, such as highlighting microvascularization in tumors.

**Figure 4:**
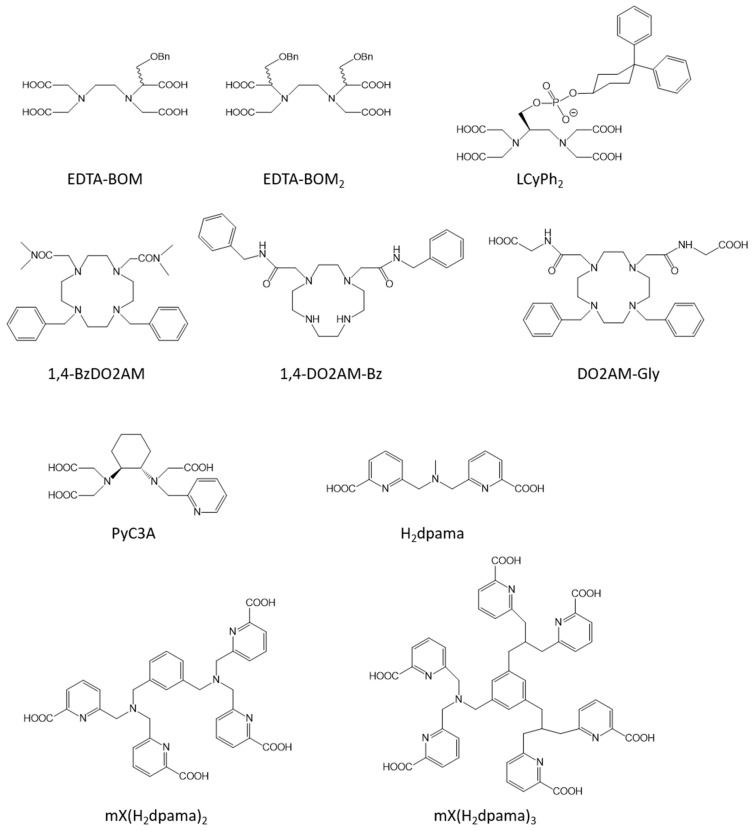
Structures of some of the ligands used for the design of the blood pool agents described in this work.

Targeting HSA (human serum albumin), one of the most abundant proteins in the blood plasma, is the most common method in the literature. HSA has two binding sites in its tridimensional structure which are known to bind organic molecules with hydrophobic moieties. Different strategies can thus be evidenced to target HSA. (i) The grafting of different hydrophobic moieties on commonly used chelates. We can cite the grafting on EDTA of one or two benzyloxymethyl (BOM) groups [72], of the same moiety as that used in MS-325 (Mn-LCyPh_2_) [73] or of deoxycholic acid [74]; the grafting of a biphenyl substituent on the ligand PC2A [75]; the grafting of benzyl groups on the 1,4-DO2AM platform (1,4-BzDO2AM, 1,4-DO2AM-Bz, and DO2AMGly) [76,77]; or the grafting on NOTA of the truncated Evans blue dye [78] (Figure 4). (ii) The chelate itself can have hydrophobic moieties able to promote binding to HSA. It is the case for Mn-PyC3A [32,33,34] (Figure 4); for the Mn complex developed by Stasiuk et al. [40]; for ligands developed by Platas-Iglesias et al. [79] containing pentadentate 6,6′-((methylazanediyl)bis(methylene)dipicolinic acid binding units able to form mono- (H_2_dpama), di- (mX(H_2_dpama)_2_), and trinuclear (mX(H_2_dpama)_3_) complexes with Mn^2+^ ions (Figure 4); or for aza-semi-crown pentadentate ligands rigidified by pyridine and piperidine rings developed by Ai et al. [80]. For all those complexes, a huge increase in the relaxivity is observed in the presence of HSA due to the formation of a non-covalent adduct (Table 1).

Another strategy consists of the development of amphiphilic paramagnetic complexes able to form micelles endowed with a high plasmatic half-life and a high relaxivity. Tei et al. [81,82] synthesized six original amphiphilic ligands based on EDTA or on DO2A grafted with aliphatic chains. A strong self-association in micelles was observed, resulting in an enhanced relaxivity. Furthermore, micelles were able to interact with HSA, increasing even more the relaxivity. In another study, PEGylated amphiphilic polymeric Mn complexes were developed and showed an enhanced relaxivity as well as an excellent and relatively long-time-window vascular enhancement effect [83,84].

### 2.4. Responsive Contrast Agents

Responsive contrast agents, also called smart or intelligent CAs, are able to report changes in a physiologically relevant parameter, such as pH, redox state, levels of some endogenous ions (Zn^2+^, Ca^2+^ or Cu^2+^), etc.

**Figure 5:**
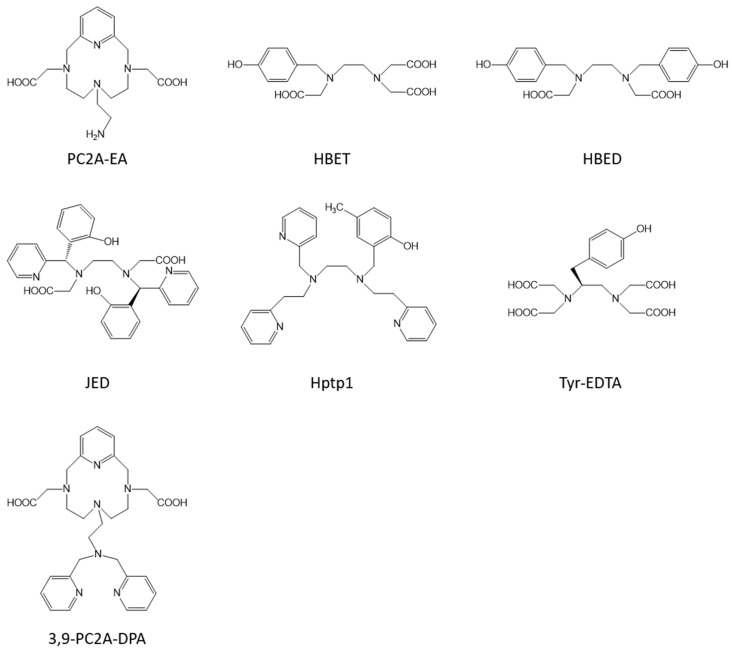
Structures of some of the ligands used to obtain responsive contrast agents.

The mapping of tissue pH could allow the diagnosis of tumors at an early stage since their enhanced glucose metabolism induces a decrease in the extracellular pH (Warburg effect) [85]. pH-responsive contrast agents will be able to evidence this pH decrease by a change in their relaxivity induced by a change in the number of coordinated innersphere water molecules. A first example is the Mn-PC2A-EA [86] with an ethylamine pendant arm (Figure 5). At acidic pH (between 3.7 and 5.8), the protonation of the amine function allows the presence of one innersphere water molecule, with a relaxivity of 3.5 s^−1^ mM^−1^ at 0.47 T and 25 °C, but when the pH increases, the deprotonation of the amine function allows its coordination to the metal, inducing the loss of the innersphere water molecule and hence a decrease in the relaxivity to 2.1 s^−1^ mM^−1^ (Table 1). Other studies have used the interesting protonation transition of sulfonamides groups around the physiological pH to construct pH-responsive contrast agents. Platas-Iglesias et al. [42] developed several complexes characterized by a transition from one innersphere water molecule at basic pH to two innersphere water moleules at acidic pH, with a relaxivity changing from 3.8 s^−1^ mM^−1^ at pH 9 (10 MHz, 25 °C) to 8.9 s^−1^ mM^−1^ at pH 4. In a more recent study involving a sulfonamide group grafted on a triazacyclononane macrocycle, Liang et al. [87] observed a change in relaxivity from 0.9 s^−1^ mM^−1^ at pH 7–9.5 (20 MHz, 25 °C) characteristic of a q = 0 complex to 3.0 s^−1^ mM^−1^ at pH 7–4.5, typical of the presence of one innersphere water molecule.

A modification of the redox status of tissues is a well-known feature of different diseases such as cancers, ischemia, or chronic inflammation. Being able to detect changes in redox activity in vivo could thus be very important in the diagnosis of those pathologies. Toward that aim, using the couple Mn(II)/Mn(III) ions can be an elegant method to monitor redox imbalance. Indeed, Mn(II) complexes are generally characterized by higher relaxivities than their Mn(III) equivalents, as explained earlier. Caravan et al. [88,89,90] largely exploited this way by developing several generations of complexes based on the EDTA core modified with one or several hydroxybenzyl moieties (HBET, HBED, and JED, Figure 5) able to form stable complexes with both Mn(II) and Mn(III) ions. The more recent system based on the JED ligand allows a 9-fold enhancement of the relaxivity when Mn(III) is reduced to Mn(II) (Table 1).

The detection of oxidative stress is also a major challenge since it is linked to tissue damage in many diseases (Alzheimer, Parkinson, atherosclerosis, etc.). Being accompanied by the production of reactive oxidative species (ROS), Mn complexes able to directly detect ROS have been developed [91,92]. Two generations were elaborated: the first one is based on an original ligand (*N*-(2-hydroxy-5-methylbenzyl)-*N*,*N*′,*N*′-*tris*(2-pyridinylmethyl)-1,2-ethane-diamine, Hptp1, Figure 5) able to form a stable complex with Mn(II) ions. Upon reaction with H_2_O_2_, the complex couples to itself to form a dimer, with a resulting decrease in the relaxivity. However, this strategy has the disadvantage that the production of ROS would be detected by a decrease in the contrast (negative contrast) [91]. A second generation was thus developed where an oxidizable quinol group is grafted on the same type of ligand. This allows to observe an increase in the relaxivity upon the presence of H_2_O_2_ (Table 1) [92].

ROS being produced notably by myeloperoxidase (MPO), a heme protein, another strategy consists of developing contrast agents of which the relaxivity is modified when this enzyme is overexpressed. This is the case for the complex Mn-Tyr-EDTA (Figure 5) where a tyrosine derivative is grafted on EDTA and which demonstrates a peroxidase activity-dependent relaxivity by forming oligomers in the presence of the enzyme, inducing an increase in the relaxivity (Table 1) [93].

Another example of a responsive Mn-based contrast agent was developed by Tircso et al. [94]. They synthesized a 3,9-PC2A derivative, grafted with a di-(2-picolyl)amine (DPA) moiety as an active arm (Figure 5), able to selectively bind Zn^2+^ ions in the co-presence of human serum albumin, with an increased relaxivity (Table 1). Moreover, this complex is characterized by a good thermodynamic stability (pMn = 8.79) and a high kinetic inertness toward zinc transmetallation (t_1/2_ at pH 6.0 = 64.5 h).

### 2.5. Multimodal Contrast Agents

Multimodal contrast agents are designed to be used in different imaging techniques. One bimodality well developed in the clinical field is the combination of MRI with PET (positron emission tomography) [95] since it allows coupling the high resolution of MRI with the high sensitivity of PET. As MRI images can be recorded without the use of any contrast agents, dual PET/MRI can be performed with single PET probes. Nevertheless, the development of dual MRI/PET probes is important because MRI images will also benefit from a contrast enhancement, which, if we refer to all the studies described before, could be tissue-specific or biomarker-responsive. The positron-emitting ^52^Mn having interesting decay properties (t_1/2_ = 5.6 d) for PET imaging, dual probes able to complex radioactive ^52^Mn and cold ^55^Mn are thus promising. This allows to overcome the major problem of combining both techniques in the same probe, i.e., the big sensitivity difference between both techniques which necessitate millimolar concentrations for MRI and nanomolar concentrations for PET. Moreover, it also guarantees that both reporter molecules are chemically identical and are hence endowed with a similar biodistribution. Neumaier et al. [96] described the development of such a probe by grafting different functional groups on the CDTA chelate. They obtained Mn complexes with good thermodynamic and kinetic stabilities as well as interesting relaxivities. Another group has developed dual PET/MRI probes based on a 3,9-PC2A derivative where one of the amine nitrogen was replaced by an etheric oxygen atom, which decreases the basicity of the ligand without affecting its stability when complexed with Mn(II) ions [97].

**Figure 6:**
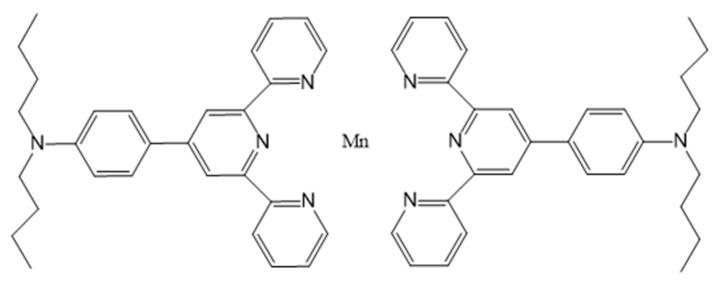
Structure of the theranostic agent FD-Mn-FD.

Another well-developed bimodality is the combination of MRI, characterized by a high resolution, and optical imaging, endowed with a good sensibility. The reporter probes for optical imaging are fluorescent molecules emitting light in the near-infrared (NIR) region to limit the absorption by the tissues. Those fluorescent molecules could be grafted on Mn-based contrast agents and it has been exploited recently by Edwards et al. who grafted hydrophobic functional groups, as chromophores, on EDTA bisamides [98]. Another study by Zhang et al. [99] describes two kinds of terpyridine–Mn(II) complexes (FD–Mn–O_2_NO and FD–Mn–FD, Figure 6) possessing seven and six coordination modes, respectively, as dual probes for multi-photon fluorescence imaging (MP-FI) and MRI. The second complex FD-Mn-FD is the most promising one, with interesting optical properties (excitation wavelength at 1450 nm (NIR-II)) and relaxometric properties (r_1_ = 2.6 s^−1^ mM^−1^ at 20 MHz and 25 °C). Moreover, this complex could also act as a therapeutic agent for the treatment of cancer by photodynamic therapy (PDT). This technique uses a photosensitizer, which, upon activation by light, can kill cancer cells. In that study, FD–Mn–FD generates endogenous ^1^O_2_ under irradiation by 808 nm light, thereby enhancing the PDT effect in vitro and in vivo.

**Table 1:** Relaxometric properties and application area of the molecular Mn complexes discussed in this work.

	q	r_1_ in Water or Buffer (s^−1^ mM^−1^)	r_1_ in the Presence of HSA (s^−1^ mM^−1^)	Application Area	Tested In Vitro and/or In Vivo
Mn-EDTA	1	2.9 (0.47 T, 35 °C, [27])		extracellular	no
Mn-CDTA	1	3.0 (0.47 T, 40 °C, [30])		extracellular	no
Mn-PyC3A	1	2.1 (1.4 T, 37 °C, [31])	3.5 (1.4 T, 37 °C, [31])	extracellular/blood pool	yes [33,34]
Mn-DPAA	1	2.7 (0.47 T, 37 °C, [35])		extracellular	no
Mn-DPAMeA	2	5.1 (0.47 T, 37 °C, [35])		extracellular	no
Mn-DPAPhA	2	4.2 (0.47 T, 37 °C, [35])		extracellular	no
Mn-PAADA	2	3.3 (0.47 T, 37 °C, [36])		extracellular	no
Mn-AMPTA	1	2.6 (0.47 T, 37 °C, [37])		extracellular	no
Mn-AMPDA-HB	1	2.7 (0.47 T, 37 °C, [37])		extracellular	no
Mn-MeNO2A	1	2.2 (0.47 T, 37 °C, [46])		extracellular	no
Mn-DO3A	0	1.3 (0.47 T, 37 °C, [49])		extracellular	no
Mn-1,7-DO2A	0	1.3 (0.47 T, 37 °C, [49])		extracellular	no
Mn-1,4-DO2A	1	1.7 (0.47 T, 37 °C, [49])		extracellular	no
Mn-1,4-DO2AM	1	2.0 (0.47 T, 37 °C, [51])		extracellular	no
Mn-AAZTA	0	1.6 (0.47 T, 25 °C, [53])		extracellular	no
Mn-AAZ3A	1	2.5 (0.47 T, 25 °C, [53])		extracellular	no
Mn-MeAAZ3A	1	2.0 (0.47 T, 25 °C, [53])		extracellular	no
Mn-AAZ3MA	1	1.9 (0.47 T, 25 °C, [53])		extracellular	no
Mn-3,6-PC2A	1	2.7 (0.47 T, 25 °C, [54])		extracellular	no
Mn-3,9-PC2A	1	2.9 (0.47 T, 25 °C, [54])		extracellular	no
Mn-15-pyN_5_	2	3.1 (0.47 T, 37 °C, [57])		extracellular	no
Mn-15-pyN_3_O_2_	2	3.6 (0.47 T, 37 °C, [57])		extracellular	no
Mn-EDTA-BTA	1	3.5 (1.5 T, 24 °C, [64])	15.1 (1.5 T, 24 °C, [64])	liver	yes [63]
Mn-EDTA-EOB	1	2.3 (1.5 T, 24 °C, [64])	6.3 (1.5 T, 24 °C, [64])	liver	yes [64]
Mn-EOB-PC2A	1	2.8 (1.5 T, 25 °C, [66])	5.9 (1.5 T, 25 °C, [66])	liver	yes [66]
Mn-NOTA-NP	1	3.6 (3 T, 25 °C, [67])	9.0 (3 T, 25 °C, [67])	liver	yes [67]
Mn-PyC3A-3-Obn	1	2.6 (1.4 T, 37 °C, [68])	9.0 (1.4 T, 37 °C, [68])	liver	yes [68]
Mn-BnO-TyEDTA	1	4.3 (0.47 T, 32 °C, [69])	15.8 (0.47 T, 32 °C, [69])	liver	yes [69,70]
Mn-EDTA-BOM	1	3.6 (0.47 T, 25 °C, [72])	55.3 (0.47 T, 25 °C, [72])	blood pool	no
Mn-LCyPh2	1	5.8 (0.47 T, 37 °C, [73])	48.0 (0.47 T, 37 °C, [73])	blood pool	yes [73]
Mn-1,4-BzDO2AM	1	3.8 (0.47 T, 25 °C, [76])	18.5 (0.47 T, 25 °C, [76])	blood pool	no
Mn-1,4-DO2AM-Bz	1	3.5 (0.47 T, 25 °C, [76])	27.4 (0.47 T, 25 °C, [76])	blood pool	no
Mn-DO2AM-Gly	1	4.5 (1 T, 25 °C, [77])	14.0 (1 T, 25 °C, [77])	blood pool	yes [77]
Mn-dpama	2	4.2 (0.47 T, 37 °C, [79])	12.2 (0.47 T, 37 °C, [79])	blood pool	no
mX(Mn-dpama)_2_	2	6.1 (0.47 T, 37 °C, [79])	39.0 (0.47 T, 37 °C, [79])	blood pool	no
mX(Mn-dpama)_3_	2	8.3 (0.47 T, 37 °C, [79])	45.2 (0.47 T, 37 °C, [79])	blood pool	no
Mn-PC2A-EA	1	3.5/2.1 (0.47 T, 25 °C, [86])		pH responsive	no
Mn^II/III^-HBET	1	1.0/2.8 (1.4 T, 37 °C, [88])		redox responsive	no
Mn^II/III^-JED	1	0.5/3.3 (1.4 T, 37 °C, [90])		redox responsive	no
Mn-Hptp1	1/2	4.7/5.3 (3 T, 25 °C, [92])		redox responsive	no
Mn-Tyr-EDTA	1	3.3/8.5 (0.47 T, 32 °C, [93])	8.0 (0.47 T, 32 °C, [93])	redox responsive	yes [93]
Mn-3,9-PC2A-DPA	1	3.2 (1.4 T, 37 °C, [94])	12.1 (1.4 T, 37 °C, [94])	Zn responsive	yes [94]

### 2.6. In Vitro/In Vivo Studies and Toxicity Issues

As shown in Table 1, only a few Mn complexes were tested in vitro and/or in vivo. Surprisingly, most of those complexes are endowed with an increased lipophilicity, allowing their use as liver-targeting contrast agents or blood pool agents. Biodistribution studies by MRI and ICP show a dual renal and hepatobiliary elimination for all those agents, which is explained by their enhanced lipophilicity compared to small extracellular agents like Gd-DOTA. Mn-PyC3A was also tested in a rat model of renal impairment and the in vivo studies indicate in that case an increased hepatobiliary elimination [34]. Moreover, Mn levels had returned to the baseline within 24 h after injection for all those complexes.

Their efficacy as MRI contrast agents was also tested. Liver-targeted contrast agents were systematically injected into a murine liver tumor model to evaluate their ability to differentiate normal liver and tumor tissue. MRI images show, for most of the complexes, a hypointense signal in tumor tissues compared to normal liver tissues after injection of the Mn-complex. This can be explained by the transport mechanism of the Mn complexes to the liver: they can enter normal hepatocytes through organic anion-transporting polypeptide transporters (OATPs) which are considerably reduced in tumor tissues. The study of Zhu et al. [69] has particularly evidenced the importance of OATPs in the hepatic uptake of Mn complexes by performing images in the presence of an OATP inhibitor as well as cell uptake studies on OATP-transfected and non-transfected cell lines. Nevertheless, the study on Mn-NOTA-NP [67] contradicts the above results since a hyperintense signal is observed in tumor tissues compared to the normal liver. The authors explain this result by decreased MRP2 expression in tumor cells whereas OATP expression is maintained. As the role of MRP2 is to mediate the secretion of the Mn complex from the tumor cells to the lumen, its decreased expression induces an accumulation of the Mn complex in the cytoplasm of tumor cells, which explains the observed hyperintense signal. Thus, it evidences the need for more thorough investigations in the future. A few blood pool agents have also been studied in vivo to evaluate their efficacy. Mn-LCyPh_2_ was injected into white rabbits at doses of 30 µmol/kg and 10 µmol/kg and good vascular images could be obtained for both doses. The authors were also able to distinguish injured from normal vessels [73]. The study on Mn-DO2AM-Gly was more focused on the ability of the Mn complex to accumulate in a highly vascularized tumor model; interesting results were obtained on subcutaneous breast tumor lesions where a strong contrast enhancement was obtained [77]. The redox responsivity of Mn-Tyr-EDTA was also evaluated in vivo on a murine model with monosodium urate crystal-induced acute gouty arthritis. The contrast enhancement in the inflammation site was higher than that obtained with Gd-DTPA used as a control.

Even if the above studies are encouraging, very few data exist about the possible toxicity of all those agents. Some of the abovementioned studies present cell viability assays [63,64,67,69,77] to evaluate the toxicity of the Mn complexes. The results showed a negligible cytotoxicity toward various cell lines in the concentration range needed for MRI. Nevertheless, it is not sufficient at all to attest to the safe use of those complexes in vivo. Indeed, as for Gd complexes, the release of free Mn^2+^ ions in vivo could be responsible for pathological disorders for patients, such as manganism, a disease with symptoms close to those of Parkinson’s disease. It is thus crucial to verify that Mn complexes remain intact when they are injected in vivo, which is nearly never the case. The study by Caravan et al. [73] on Mn-LCyPh_2_ mentions that the complex should remain intact since no acute cardiac toxicity was evidenced during their study and free Mn^2+^ ions are known to be very toxic for the heart. This is nevertheless indirect proof so that more thorough studies, such as those performed on gadolinium complexes when concerns about NSF and gadolinium retention in the brain start to appear, are needed to attest to the safety of all those Mn complexes. Scientists must take advantage of the knowledge acquired about gadolinium complexes to avoid repeating the same mistakes and develop newer and safer MRI contrast agents.

## 3. Nanoparticular Contrast Agents

### 3.1. Nanoparticles Incorporating Mn Complexes

The aforementioned Mn complexes are globally characterized by limited relaxivities and an elegant manner to increase their efficacy is an increase in their rotational correlation time τ_R_, even by an increase in their molecular weight or by their incorporation in nanosystems. This has been largely exploited in the literature.

As described previously [81,82,83,84], amphiphilic Mn complexes can be assembled in lipidic nanoobjects, such as micelles or liposomes, with considerable relaxivities. For example, Ai et al. [84] co-assembled amphiphilic Mn chelates (C18-PhDTA-Mn) with amphiphilic PEG-C18 polymers to obtain mixed micelles of different hydrodynamic sizes and relaxivities up to 13 s^−1^ mM^−1^ at 1.5T and 25 °C.

Dendrimeric nanosystems are another well-exploited method to increase the rotational correlation time of Mn complexes. Lu et al. [100,101] describe the grafting of Mn(II)-DOTA monoamides on lysine dendrimers with a silsesquioxane core. Different generations (G2, G3, and G4) of dendrimers were synthesized, with a decrease in the per ion relaxivity when the generation of the carriers increases. This can be explained by the lack of any innersphere water molecule on the Mn-DOTA complexes, as explained previously. The relaxivity thus only comes from a secondary solvation sphere due to hydrogen bonding between water and Mn complexes and from a less organized outer solvation sphere. As the dendrimer generation increases, the probability of hydrogen bonding also increases, which could increase the residence time of water molecules in the secondary sphere and have a detrimental effect on the relaxivity. Moreover, DOTA chelates could be buried in the higher generations and be less accessible to solvation. The authors also tried to replace DOTA by NOTA and they obtained a better relaxivity thanks to the presence of one innersphere water molecule. More recently, Gao et al. [102,103] described three generations of DOTA-branched organic frameworks constructed by uniting DOTA building blocks. They carefully characterized the kinetic inertness of their systems and obtained an inertness 69-fold higher than that of Magnevist. This could be explained by three factors: (i) the chelates in the macromolecular structure are considerably “squeezed”, which hinders the release of Mn^2+^; (ii) the positively charged core of the nanosystems repels protons and other positively charged ions, increasing the kinetic inertness; and (iii) the metal ions released from the dendrimers could be recaptured by the numerous chelating groups, increasing the time needed to completely release Mn^2+^. We can also cite the work of Caravan et al. [28], already described in a previous section, who grafted six tyrosine-derived EDTA moieties on a cyclotriphosphazene core.

The incorporation of Mn complexes inside nanosystems is also an interesting path to explore. Botta et al. [104] incorporated Mn-CDTA bisamides complexes in nanogels based on a chitosan matrix. The Mn complexes were covalently grafted on chitosan and acted as contrast media and as cross-linking agents. They obtained relaxivities seven times higher than those of small Mn complexes thanks to the restricted mobility of the complex combined with a fast exchange of the innersphere water molecule. Moreover, the stability at physiological pH is very good. Another study explores the non-covalent encapsulation of a hexadentate pyridine-picolinate Mn complex within a porous silica nanosphere. The entrapped complex exhibits a relaxivity at 25 °C and 1.41 T 2.9 times higher than that of the unentrapped complex. The kinetic inertness toward Zn^2+^ ions and physiologically relevant anions (bicarbonates, biphosphonates, and citrate) is also very good [105]. Axelsson et al. [106,107] developed an organophosphosilane hydrogel with strongly-chelated manganese (II) ions and a covalently attached PEG surface layer. This nanosystem has a globular shape, an average hydrodynamic diameter of 5 nm, and a relaxivity of 30 s^−1^ mM^−1^ at 1.41 T and 25 °C. It is currently being evaluated in a Phase IIa clinical trial as an open-label proof-of-concept study evaluating its safety and MRI-enhancing properties in adult female patients with suspected endometrial lesions. (NCT05664828).

Mn complexes can also be grafted at the surface of inorganic nanoparticles such as silica nanoparticles. Mn-DTPA derivatives were grafted at the surface of mesoporous silica nanoparticles, with a good relxivity of 7.18 s^−1^ mM^−1^ at 25 °C and 1 T [108]. Nevertheless, even if the porous structure provides easy access for water protons, the enhanced relaxivity is limited by the fact that there is no innersphere water molecule on the DTPA chelates. In a more recent study, Mn(II)-CDTA derivatives were grafted onto silica nanoparticles [109]. The obtained relaxivity at 1 T and 25 °C is higher (around 12 s^−1^ mM^−1^) than for the previous study thanks to the presence of one innersphere molecule.

### 3.2. Mn-Based Organic/Inorganic Nanoparticles

Mn ions can also be incorporated in the structure of the nanoparticles and a lot of examples are available in the literature. Nanoparticles indeed have a lot of advantages such as the tunability of their size and shape, a high surface-to-volume ratio, and the possibility to easily functionalize them to obtain targeted, multimodal, or theranostics agents [110,111]. Even if a comprehensive review of those systems extends beyond the scope of this viewpoint, the most relevant examples are cited below.

Manganese oxide nanoparticles (MONs), such as MnO, MnO_2_, and Mn_3_O_4_, are well studied as T_1_ MRI contrast agents [112,113,114] and, more particularly, MnO nanoparticles as the oxidation state of Mn is +2, assuring five unpaired electrons and hence a better efficacy to increase water T_1_ relaxation rate. Owing to the efficacy coming from the Mn ions at the surface, two crucial factors to have a high relaxivity can be highlighted: (i) the size of the nanoparticles, which has to be as small as possible to enhance the surface to volume ratio, and (ii) the coating used to stabilize the nanoparticles, which has to be as hydrophilic as possible to assure a good penetration of water. Moreover, MnO nanoparticles can be retained by the reticuloendothelial system (RES) and subsequently accumulate in liver and spleen, leading to Mn^2+^-induced toxic effects. The coating thus has to be cleverly chosen in order to limit the capture by the RES. Different coatings have been tried in the literature such as polymer functionalization [115,116,117] (and particularly polyethylene glycol (PEG) coating), silica coating [118], and phospholipid modification [119]. Even if all those nanosystems can accumulate passively in tumors by the EPR effect, some researchers also added specific targeting ligands (such as aptamers or cRGD peptide) to increase the uptake by the tumor over a long period of time [115,116,120]. T_1_–T_2_ dual mode contrast agents, obtained by combining MnO (as T_1_ agent) and Fe_3_O_4_ (as T_2_ agent), have also been extensively studied [121,122]. The bimodality can also come from another imaging technique, such as optical imaging (OI), which is a more sensitive imaging technique than MRI, as explained in a previous section. MnO nanoparticles can easily be functionalized by near-infrared dyes, such as Cy5.5, as a contrast agent for OI [123,124].

Besides the development of manganese oxide nanoparticles, Mn ions have also been incorporated in other types of inorganic nanoparticles. We can cite the incorporation of Mn ions in cyano-bridged coordination networks, such as Prussian blue nanoparticles [125], the grafting of manganese ions on the surface of nanodiamonds [126,127], the encapsulation of Mn^2+^ ions in sealed carbonized shells [128], and the development of carbon dots doped with manganese [129,130]. This last system has the advantage of also being detectable in optical imaging since carbon nanodots are luminescent. All those described systems are characterized by higher relaxivities than the small Mn complexes described in Section 2 and are thus good candidates as T_1_ MRI contrast agents. Dual T_1_/T_2_ agents are also developed. Zhang et al. [131] designed ferroferric oxide coated by Mn-doped silica as an intelligent MRI nanoswitch. Under normal tissue conditions, the nanostructure is very stable, so the r_1_ and r_2_ relaxivities are very low but the slightly acidic pH of tumors is responsible for the nanostructure collapse, releasing the Mn^2+^ ions, which are separated from the Fe_3_O_4_ magnetic core, with a resulting increase in the relaxivities r_1_ and r_2_.

Organic nanoparticles are also well exploited in the literature to incorporate manganese ions. More particularly, metal–organic frameworks (MOFs), where a small ligand and a metal center are alternatively linked together to form a porous structure with a defined shape, have attracted much attention. For example, Aoki et al. [132] developed Mn-MOF-74, where the ligand dihydroxyterephthalate (DHTP) self-assembles with Mn^2+^ ions to form a honeycomb-like structure with a diameter of 1.0 nm. They obtained an interesting relaxivity of about 10 s^−1^ mM^−1^ at 1.0 T and 23 °C. Zhao et al. [133] developed a Mn(II)-chelated ionic covalent organic framework (iCOF) and also obtained an interesting relaxivity (8 s^−1^ mM^−1^ at 3.0 T and 25 °C). Natural organic particles can also be used, such as proteins, and this has notably been exploited recently by Colombo et al. [134] who loaded Mn ions in H-ferritin, a recombinant variant of human apoferritin consisting of 24 self-assembled heavy-chains subunits. Two formulations were prepared, at room temperature and at 65 °C, with a very low relaxivity for the latter compared to the formulation at room temperature. This was explained by the oxidation of the Mn ions to Mn^3+^, Mn^4+^, and Mn^7+^ in the formulation obtained at high temperature.

### 3.3. In Vitro/In Vivo Tests and Toxicity Issues

*Nanoparticles incorporating Mn complexes:*

Most of the presented systems were studied in vivo to evaluate their efficacy as MRI contrast agents. Overall, they are eliminated through the kidneys or the liver according to their size and they are also able to accumulate in tumors and/or lymph nodes due to the enhanced permeability and retention effect (EPR). This effect is based on the abnormalities that appear in the tumor microenvironment. Indeed, tumors exhibit poor lymphatic drainage and vessels with a higher permeability than healthy vessels. These two points allow accumulation of the nanostructures in the tumor site, which could favor tumor diagnosis.

Nevertheless, as already mentioned in the previous section concerning the small Mn complexes, very few toxicity data are available. Again, some studies present encouraging cytotoxic studies on various cell lines [84,103,104,108] which do not show any acute toxic effect. We can also cite the study of Ai et al. [83], who measured key serum biochemical indicators of liver function and kidney function and found a normal range for all of them. Gao et al. [103] also evidenced the absence of tissue injury or inflammation in any of the major organs two days after the injection of their dendrimeric nanosystems. All of those indicators are very encouraging but more systematic studies of the toxicity are still missing.

*Mn-based organic/inorganic nanoparticles:*

Those nanoparticles were almost all tested in vitro and in vivo and exhibit overall interesting properties as T_1_ MRI agents. Cytotoxic studies on various cell lines indicate globally good cell viability for most of the tested systems; most of the in vivo studies do not report any pathological abnormalities through hematoxylin and eosin (H&E) staining of the major organs (heart, kidney, liver, lung, and spleen). This is thus encouraging and suggests that most of the cited nanosystems have a low cytotoxicity and good biocompatibility. Nevertheless, as already mentioned for the previous systems, more systematic studies of the toxicity of those nanoparticles are needed. At the moment, all the studies are focused on the performance of the systems as MRI contrast agents but a translation to the clinic will necessitate the establishment of a transparent cytotoxicity pattern.

## 4. Theranostic Agents

Mn-based theranostic agents, combining an MRI contrast agent based on manganese and a therapeutic tool, are increasingly developed in the literature, especially in the field of cancer diagnosis and treatment. Only a few molecular examples are described. Additionally to the study by Zhang et al. [99] (see previous section), a salinomycin-based paramagnetic complex of manganese was synthesized. To overcome its water insolubility, it was loaded into empty bacterial ghosts (BGs) cells as transporters. Its relaxivity is similar to that reported for small Mn complexes and it was able to induce perturbations in the cell cycle of colorectal and breast human cancer cell lines [135]. Nevertheless, most of the research concerns nanoparticular agents since it is quite easy to modify their surface to incorporate several functional moieties [136,137,138]. Even if a detailed description of all the developed systems is beyond the scope of this review, we can cite some recent relevant examples.

Nanoparticular agents can be divided into two categories:

(i) Nanoparticles incorporating a drug (such as doxorubicin, cisplatin, paclitaxel, or docetaxel) able to kill cancer cells. Different studies are available in the literature. For example, the development of polymeric micelles or vesicles (polymersomes) incorporating doxorubicin is described. Alibolandi et al. [139] described the development of polymersomes based on an amphiphilic diblock copolymer of poly(ε-caprolactone)-block-poly(glyceryl methacrylate) encapsulating doxorubicin in the hydrophilic core of the vesicles and a hydrophobic Mn complex based on phenanthroline in the bilayer membrane. Moreover, an aptamer was added on the surface to increase the specificity of the nanosystem for colorectal cancer cells. Another study designed a nanosystem based on a hydrophobic core made of doxorubicin complexed with Mn^2+^ ions stabilized by an outer layer composed of a self-assembled amphiphilic block copolymer distearyl phosphatidylethanolamine-polyethylene glycol (DPSE-mPEG2000) [140]. Both systems show good therapeutic and MRI abilities to treat and monitor tumors. Inorganic nanoparticles are also developed. As an example, the design of pH-responsive Mn-ZnO nanoparticles was recently described. The nanoparticles are coated with polyacrylic acid and decorated with folic acid to target tumor cells. Doxorubicin is loaded into the system through electrostatic interactions with polyacrylic acid and can be released from the nanocomposite at slightly acidic pH [141];

(ii) Nanoparticles including other therapeutic tools, such as photodynamic therapy (PDT) or photo–thermal therapy (PTT), which are both light-excited treatment prototypes. PDT induces the production of reactive oxygen species (ROS) through the light activation of photosensitizers whereas PTT uses photothermal conversion agents to generate heat and kill cancer cells. The design of Mn-doped magnetosomes was reported recently [142]. They are naturally produced through the biomineralization of magnetotactic bacteria by doping manganese into the iron oxide crystals. The further functionalization of the surface with cRGD peptides allows accumulation in tumors. This agent can act as a dual T_1_/T_2_ MRI agent and has also a good optical absorbance in the NIR region, leading to a high photothermal capability. Another study is using rod-like cellulose nanocrystals coated with crosslinked polydopamine as photothermal agents, and Mn^2+^ ions were embedded into the crosslinked polymeric coating as MRI contrast agents. Both an interesting relaxivity and photothermal conversion efficiency of 38 s^−1^ mM^−1^ (at 3T and 25 °C) and 44% (after irradiation at 808 nm with an output power of 2 W·cm^−2^ during 12 min) were, respectively, obtained [143]. Smart systems, avoiding side effects in normal tissues, are also increasingly developed. Zhao et al. [144] designed a switchable nanosystem that can act as a ROS scavenger in normal tissues and as a ROS generator in the tumor microenvironment during PDT. It is based on a Mn_3_O_4_ nanozyme coated with 1,2-distearoyl-sn-glycero-3-phosphoethanolamine-*N*-[amino(polyethylene glycol)-2000] (DSPE-PEG_2000_ amine) to insure the solubility in water and loaded with pyropheophorbide as a PDT agent. This nanosystem is able to down-regulate ROS with a dampened cytokine wave in normal tissues after PDT whereas in the tumor microenvironment, glutathione (GSH), which is known to be overexpressed in cancer cells, will degrade the nanosystem, increasing the production of ^1^O_2_. Moreover, the oxidative stress of the tumor tissue will be increased through the liberation of Mn ions during the degradation. They are indeed able to produce cytotoxic hydroxyl radicals (^●^OH) via a Fenton reaction with H_2_O_2_. Additionally, the released Mn ions show a strong T_1_ MRI contrast.

Chemodynamic therapy (CDT) is also increasingly developed. It is based on the principle described above, i.e., the production of cytotoxic hydroxyl radicals (^●^OH) via a Fenton reaction with H_2_O_2_. Mn-based nanoparticles are particularly useful in that case, especially MnO_2_ nanoparticles, since Mn^4+^ ions can be reduced to Mn^2+^ by GSH. The produced Mn^2+^ ions can then enhance T_1_-MRI but also react with H_2_O_2_ by a Fenton reaction to produce hydroxyl radicals (^●^OH). Those nanoparticles are thus good candidates as theranostic agents and this was well exploited in the literature, where a lot of different morphologies are described such as core-shell, hollow-spherical, Janus, nanoflower, honeycomb, or nanosheet structures [136]. For all these hybrid nanoparticles, the MnO_2_ nanoparticles are incorporated in other materials or their surface is modified to assure their stability and to optimize their efficacy. Purely inorganic materials are described, such as graphitic carbon nitride-manganese oxide nanoflowers (g-C_3_N_4_/MnO_2_) [145], but hybrid inorganic/organic nanosystems are also very well-developed. For example, Wang et al. [146] employed poly(lactic-co-glycolic acid) (PLGA) nanoparticles as a template to synthesize hollow MnO_2_. In the acidic tumor microenvironment, the fast degradation of hollow MnO_2_ nanoparticles induces the liberation of Mn^2+^ ions, catalyzing the transformation of H_2_O_2_ to ^●^OH for CDT. In this study, bufalin was also incorporated in the nanosystem as a chemotherapeutic drug released during the degradation of the hollow MnO_2_ nanoparticles.

**Figure 7:**
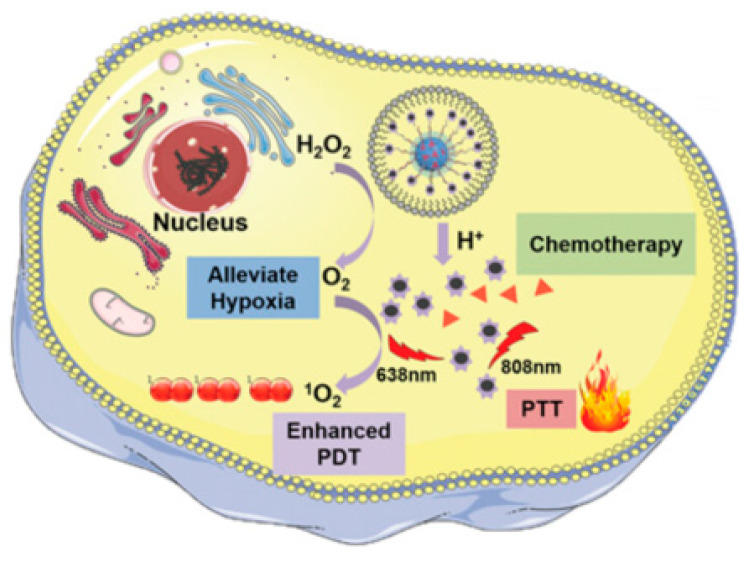
Illustration of the combination therapy using PTT, PDT, and chemotherapy to enhance tumor treatment (reproduced with permission from [149]; copyright 5634210108465).

A substantial number of studies tried also to combine several therapeutic tools in the same nanoobject (combination therapy) to increase the chances of killing the tumor cells. The combination between PTT and/or PDT with CDT or with the use of a chemotherapeutic drug, the combination between CDT and the use of a chemotherapeutic drug, or, in some studies, the combination between all those techniques, are often reported. As examples, we can cite the combination of CDT with the use of paclitaxel as a chemotherapeutic drug [147]. The nanoparticles are made of modified dopamine (DOPA)-β-cyclodextrin (CD) combined with MnO_2_-loaded nanoparticles. The surface was conjugated with the peptide tLyP-1 to increase their capacity to pass the blood–brain barrier (BBB) and reach glioma cells. As expected, the MnO_2_ core responded to H_2_O_2_ in the acidic tumor environment by releasing Mn^2+^ ions, as T_1_ MRI agent and ROS generator. Moreover, the released paclitaxel also participates in the destruction of cancer cells. In another study, the coating of gold nanorods with SiO_2_ and MnO_2_ enabled a shift in the optical absorbance of the nanocomposite from NIR-I to NIR-II, favoring PTT. Moreover, the released Mn^2+^ ions in the acidic environment of the tumor allow treatment with CDT. Additionally, MRI is combined with photoacoustic imaging in this nanocomposite [148]. As a last example, the recent study of Liu et al. [149] is very promising as it combines all the above-cited therapeutic tools in the same nanoobject, composed of hollow mesoporous MnO_2_ nanoparticles coated with poly(allylamine hydrochloride) (PAH) and poly(acrylic acid). These were subsequently covalently grafted with pegylated phosphorous quantum dots and then loaded with doxorubicin. In the tumor microenvironment, the structure is degraded, releasing doxorubicin and the phosphorous quantum dots as active species. Doxorubicin acts as a chemotherapeutic drug and as a fluorescence imaging agent, whereas the phosphorous quantum dots allow PDT and PTT under laser irradiation at 630 and 808 nm. Moreover, MnO_2_ affords an MRI contrast and facilitates the conversion of H_2_O_2_ to oxygen, enhancing PDT (Figure 7).

Toxicity issues

All of the safety concerns raised in the previous section on nanoparticle-based MRI agents remain valid in this case. But, it has to be noted that most of the above-cited examples imply the release of free Mn^2+^ ions, which, as already mentioned, could induce toxicity issues, and this is not very well studied. All the in vivo studies have shown the absence of toxicity of the Mn nanoparticles at a certain concentration, suggesting low cytotoxicity and good biocompatibility, but they could become more toxic at a higher concentration. Moreover, the long-term effects of exposure to Mn nanoparticles still have to be studied. The released Mn^2+^ ions could indeed enter the traffic routes of biological Mn, which could have latent effects. A translation to the clinic of all the above-cited examples will thus require meticulous studies of their biosafety.

## 5. Conclusions

This work highlights the numerous studies performed to design highly efficient MRI contrast agents based on manganese as a hopefully less-toxic alternative to the actually-used gadolinium complexes. Both molecular agents and nanoparticular agents are developed. For the first ones, relaxivities similar or higher to these of gadolinium complexes were obtained but special attention to the thermodynamic stability and the kinetic inertness has to be paid. Some of the described studies are particularly interesting in that sense, and we could hope a translation in the clinic in a near future. Concerning nanoparticular agents, their development is also very interesting since very high relaxivities can be obtained and they can be easily functionalized with different functional moieties. Meticulous toxicity studies will nevertheless be needed to translate those systems to the clinic. This is also true for theranostic agents which are very promising to monitor and treat tumoral tissues. Hopefully, the future will see the transfer to the clinic of many of these agents.

## Data Availability

Not applicable.
